# Utility of syndromic surveillance for the surveillance of healthcare-associated infections in resource-limited settings: a narrative review

**DOI:** 10.3389/fmicb.2024.1493511

**Published:** 2024-10-21

**Authors:** Herman Mwanja, J. P. Waswa, Reuben Kiggundu, Hope Mackline, Daniel Bulwadda, Dathan M. Byonanebye, Andrew Kambugu, Francis Kakooza

**Affiliations:** ^1^Centres for Antimicrobial Optimization Network (CAMO-Net), Infectious Diseases Institute, Makerere University College of Health Sciences, Kampala, Uganda; ^2^Management Sciences for Health, Kampala, Uganda; ^3^Global Health Security Department, Infectious Diseases Institute, Makerere University College of Health Sciences, Kampala, Uganda; ^4^Department of Medical Microbiology, Makerere University College of Health Sciences, Kampala, Uganda; ^5^Makerere University School of Public Health, Kampala, Uganda

**Keywords:** healthcare-associated infections (HCAIs), syndromic surveillance, resource-limited settings, emergency department, public health surveillance

## Abstract

Globally, Healthcare-associated infections (HCAIs) pose a significant threat to patient safety and healthcare systems. In low- and middle-income countries (LMICs), the lack of adequate resources to manage HCAIs, as well as the weak healthcare system, further exacerbate the burden of these infections. Traditional surveillance methods that rely on laboratory tests are cost-intensive and impractical in these settings, leading to ineffective monitoring and delayed management of HCAIs. The rates of HCAIs in resource-limited settings have not been well established for most LMICs, despite their negative consequences. This is partly due to costs associated with surveillance systems. Syndromic surveillance, a part of active surveillance, focuses on clinical observations and symptoms rather than laboratory confirmation for HCAI detection. Its cost-effectiveness and efficiency make it a beneficial approach for monitoring HCAIs in LMICs. It provides for early warning capabilities, enabling timely identification and response to potential HCAI outbreaks. Syndromic surveillance is highly sensitive and this helps balance the challenge of low sensitivity of laboratory-based surveillance systems. If syndromic surveillance is used hand-in-hand with laboratory-based surveillance systems, it will greatly contribute to establishing the true burden of HAIs in resource-limited settings. Additionally, its flexibility allows for adaptation to different healthcare settings and integration into existing health information systems, facilitating data-driven decision-making and resource allocation. Such a system would augment the event-based surveillance system that is based on alerts and rumours for early detection of events of outbreak potential. If well streamlined and targeted, to monitor priority HCAIs such as surgical site infections, hospital-acquired pneumonia, diarrheal illnesses, the cost and burden of the effects from these infections could be reduced. This approach would offer early detection capabilities and could be expanded into nationwide HCAI surveillance networks with standardised data collection, healthcare worker training, real-time reporting mechanisms, stakeholder collaboration, and continuous monitoring and evaluation. Syndromic surveillance offers a promising strategy for combating HCAIs in LMICs. It provides early warning capabilities, conserves resources, and enhances patient safety. Effective implementation depends on strategic interventions, stakeholder collaboration, and ongoing monitoring and evaluation to ensure sustained effectiveness in HCAI detection and response.

## Introduction

Healthcare-associated infections (HCAIs) are of significant public health concern worldwide, impacting patient safety during hospitalisation and healthcare systems in high, middle, and low-income countries (Haque et al., 2018; Maki and Zervos, 2021). These infections result in longer hospital stays, long-term disability, increased microbial resistance to antimicrobials, higher healthcare costs, financial burdens for patients and families, and unnecessary deaths (Ling et al., 2015; Marchetti and Rossiter, 2013). The burden is particularly high in low- and middle-income countries (LMICs) due to limited resources, weak health systems that include deficiencies in trained personnel, and inconsistent surveillance (Maki and Zervos, 2021; Abubakar et al., 2022; Vandijck et al., 2013).

Like many LMICs in sub-Saharan Africa, Uganda faces a daunting challenge in monitoring and managing HCAIs. Research conducted over a decade ago in two hospitals reported a prevalence of HCAIs at 34%, with the majority being mixed infections, including bloodstream infections, surgical wound infections, urinary tract infections, lower respiratory tract infections, and gastrointestinal infections (Ankunda et al., 2010; Greco and Magombe, 2011). In most cases, these infections were associated with longer hospital stays, intravenous and urinary catheterization, emergency surgeries, inadequate hand hygiene, poor isolation practices, and inadequate supplies for disinfection. These factors compromise infection prevention and control practices on the wards (Greco and Magombe, 2011). Data providing a clear national picture of HCAI surveillance in Uganda and many LMICs is limited due to the inadequacy of the traditional surveillance methods which require sophisticated laboratory and diagnostic capacity to identify the specific pathogens responsible for these infections (Haque et al., 2018).

## Public health surveillance

**Figure 1:**
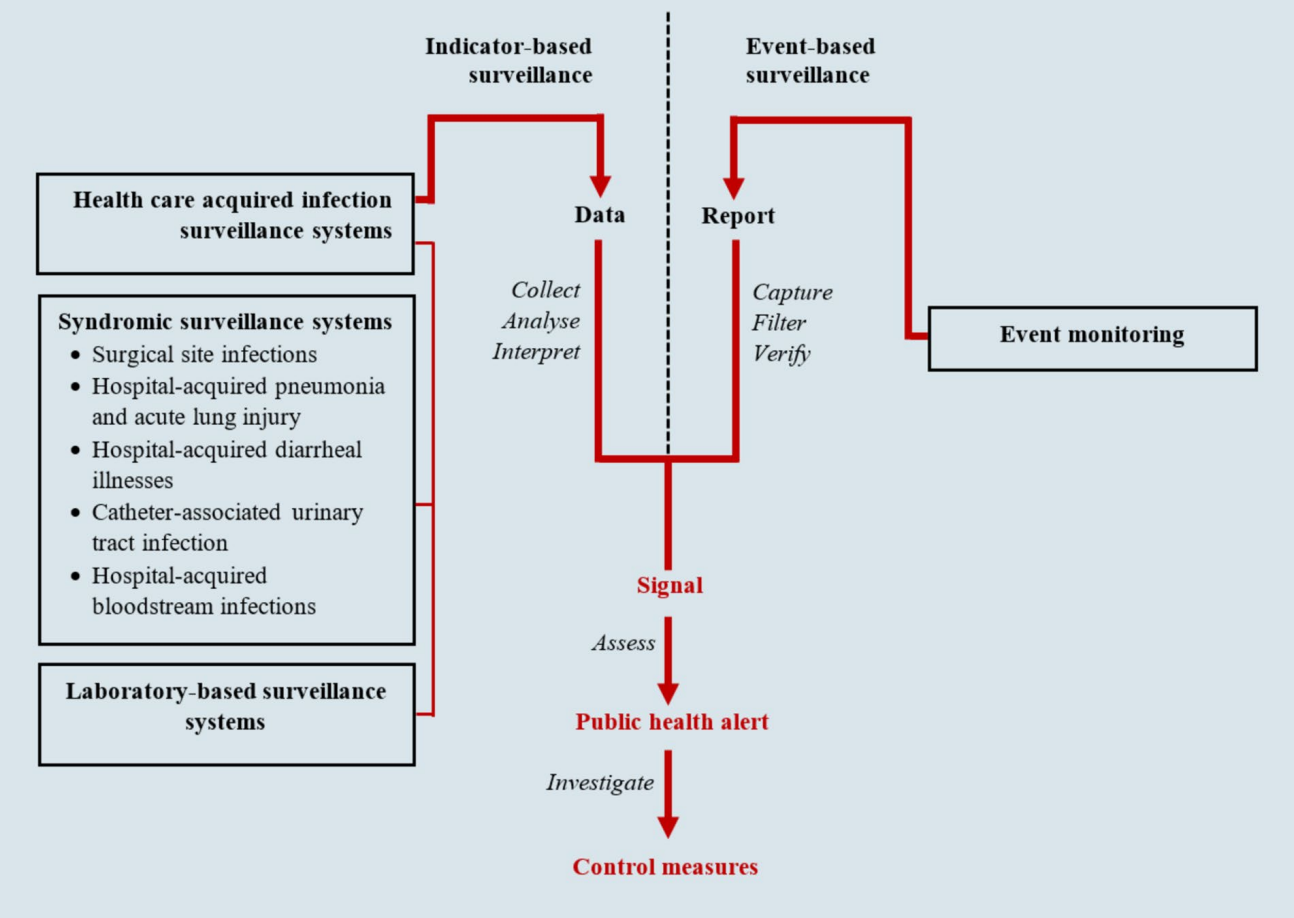
Infectious disease surveillance framework (adapted from National Academies of Sciences, Engineering, and Medicine) (Ministry of Health in Uganda, 2021; National Academies of Sciences, Engineering, and Medicine, 2007).

Public Health Surveillance is the epidemiological foundation of global health and involves ongoing systematic identification, collection, collation, analysis, interpretation, and dissemination of disease occurrence and public health event data for timely and robust action (Nsubuga et al., 2011; Berkelman et al., 2009; Ministry of Health in Uganda, 2021). It is an essential tool for measuring disease burden, including monitoring morbidity/mortality trends, to effectively guide the planning, implementation, monitoring, and evaluation of control programs and the corresponding allocation of resources (Nsubuga et al., 2011). Public health surveillance is hinged on two approaches, including indicator-based (passive) and event-based (active) surveillance as shown in Figure 1 (Ministry of Health in Uganda, 2021). Indicator-based surveillance is a system where a health jurisdiction or institution receives routine reports from hospitals, clinics, public health units, communities, or other sources. It is a relatively cost-effective approach for covering large areas, utilizing several critical indicators embedded in health management information systems to monitor diseases and outbreaks within the community (Ministry of Health in Uganda, 2021; Sharp et al., 2015). Event-based surveillance, on the other hand, involves proactive search for information about public health events, risks, or conditions from health providers, health facilities and the communities. This includes records review by health workers, screening for specific outbreak-prone health conditions such as Ebola virus disease, contact tracing during outbreaks, regular communication, and maintaining contact with key reporting sources (Ministry of Health in Uganda, 2021; Chung et al., 2015; McNamara, 2016). In both approaches, syndromic surveillance is typically applied, utilizing definitions based solely on clinical features without confirmatory laboratory diagnoses. For example, during a measles outbreak, any child below five with a rash would be considered a measles patient (May et al., 2009). This method aids in the early identification of illness clusters before diagnoses are confirmed, ensuring timely reporting to public health agencies for rapid response, thereby reducing morbidity and mortality (Henning, 2004). The two approaches complement each other. In many surveillance systems, they have been merged into integrated systems, utilizing the same infrastructure to gather information on multiple public health events of interest (Nsubuga et al., 2011). These integrated surveillance systems provide an early warning system for early detection of public health events for rapid detection, investigation, and response to public health events (World Health Organization, 2014).

## Surveillance of HCAIs

Surveillance of HAIs serves as a cornerstone for infection control programs, antimicrobial stewardship programs, patient safety and early warning systems for disease outbreak detection (Shenoy, 2023). Additionally, conducting surveillance for HCAIs allows for the detection of variations in the rate or distribution of HCAIs, which can subsequently lead to the identification of potentially contagious disease threats within the health facility (Shenoy, 2023; Surveillance CNI, 2023). For example, it could indicate hospital environmental contamination and lead to corrective action (Surveillance CNI, 2023).

### Laboratory surveillance of HCAIs

Traditional surveillance for HCAIs typically involves laboratory confirmation, active case finding, and reviewing of patient medical records during hospitalisation (Magill et al., 2015; Monegro et al., 2017; Dramowski et al., 2017; Hearn et al., 2017). Laboratory confirmation involves culturing (bacterial growth and enumeration) and antimicrobial sensitivity testing of specific pathogens from priority clinical specimens, such as blood, urine, and pus, to track infections acquired during healthcare (Dramowski et al., 2017). This confirmation is usually complemented by active case finding, where healthcare personnel actively search for patients who may have been exposed to those with the given HCAI in the wards. In some instances, healthcare personnel also review patient medical records for any signs and symptoms suggestive of infection, alongside laboratory results, to support the HCAI diagnosis (Hearn et al., 2017). Traditional HCAI surveillance is a vital tool, especially in high-income countries with adequate healthcare infrastructure. However, it is resource-intensive and involves long turnaround times, posing significant challenges in resource-limited settings such as in Sub-Saharan Africa (Maki and Zervos, 2021). consequently, alternative approaches, such as syndromic surveillance, are necessary for monitoring HCAIs in these resource-limited settings. This paper explores the use of syndromic surveillance for monitoring HCAIs in resource-limited settings that lack advanced laboratory diagnostics and real-time surveillance capabilities, such as Uganda.

### Syndromic surveillance of HCAIs

Rather than relying on laboratory confirmation of specific pathogens, syndromic surveillance focuses on identifying groups/sets of clinical signs and symptoms (syndromes) suggestive of possible infections, frequency (such as the rate of the episodes) in which these syndromes occur in a given population (Reingold, 2003; Katz et al., 2011; Boyle and Sparks, 2022). Syndromic surveillance is a fundamental approach to detecting aberrations in health data and signalling the onset of public health threats (May et al., 2011). Its core principle is to provide an early warning system that monitors disease patterns and tracks the emergence of unusual signs and symptoms grouped into case definitions. This enables public health authorities to initiate timely and effective interventions (May et al., 2011; Sosin, 2003). For HCAIs, syndromic surveillance tracks groups of symptoms or clinical signs without needing aetiological or pathological confirmation. This approach effectively bridges precise case definitions with infection rate reporting and measurement uncertainty, helping to avoid systematic undercounting of cases (Furness, 2016a,b).

## Role of syndromic surveillance of HCAIs in resource-limited settings

Unlike traditional surveillance approaches that rely on laboratory diagnostics as a starting point for HCAI surveillance, syndromic surveillance offers distinct advantages in resource-limited settings (Shenoy and Branch-Elliman, 2023).

In settings where laboratory diagnostic capacities are underdeveloped, syndromic surveillance reduces the need for extensive testing of each suspected case, thereby easing the burden on laboratories (May et al., 2011). This, in the long run, conserves resources and enables prioritized testing for confirming and characterizing outbreaks in health facilities. Additionally, monitoring syndromes rather than confirmed diagnoses allows for more efficient resource allocation based on observed syndromes, even before specific pathogens are identified (European Commission, 2012). By targeting surveillance efforts on patients at higher risk for developing HCAIs, syndromic surveillance may enhance the efficacy and cost-effectiveness of surveillance systems. Patients at increased risk may have specific risk factors such as type of disease, disease severity, medical interventions being utilised (e.g., indwelling catheters, central lines, ventilators) or because the patients are in specific places in the hospital (e.g., ICU, PICU, high-dependency units etc.) (Shenoy, 2023; Puro et al., 2022).

Syndromic surveillance offers a health facility-based early warning system for potential HCAI outbreaks by identifying suspect cases and unusual infection patterns through clusters of symptoms before laboratory confirmation is available (Nsubuga et al., 2011). This approach facilitates earlier identification, detection, and response to outbreaks, allowing for more timely interventions.

Syndromic surveillance systems’ flexibility and adaptability allow for tailoring to the specific needs and resources of a particular setting, from the highest to the lowest levels of the healthcare system where patients are admitted for care (Nsubuga et al., 2011). Thus, syndromic surveillance can be adapted and scaled to monitor various syndromes in health facilities, including those caused by yet-to-be-determined pathogens or those with non-specific symptoms.

Syndromic surveillance can be integrated into the existing healthcare systems, such as national health information systems like electronic medical records and other reporting systems such as the District Health Information System (DHIS-2) platform and the electronic Integrated Disease Surveillance and Response (e-IDSR) (Ministry of Health in Uganda, 2021). This integration can facilitate real-time monitoring of potential HCAIs based on syndromes, enabling prompt response to emerging threats.

The data generated from syndromic surveillance of HCAIs can be utilised for decision-making at various levels of the healthcare system, from individual point of care to public and global health policy (Haque et al., 2020). This data-driven approach enhances the generation of targeted interventions, including quality improvement projects and more informed resource allocation strategies.

Lastly, laboratory-based methods for hospital infection surveillance often lead to systematic underreporting (low sensitivity, high specificity). The low sensitivity, may lead to underreporting of the true burden of HCAIs and limit the ability to implement interventions aimed at reducing HCAIs effectively (Sim et al., 2024; Furness, 2016c). On the other hand, syndromic surveillance is highly sensitive helping to address this issue (Furness, 2016c). Hence, syndromic surveillance should complement laboratory surveillance, to support HCAIs control programs.

## Priority syndromes for HCAI surveillance

Table 1 shows examples of syndromes for HCAI surveillance.

**Table 1:** Examples of syndromes for healthcare-associated infections (HCAI) surveillance.

HCAI	Definition of the diagnostic syndromes	An example of a surveillance system using the syndrome
Surgical site infections	Signs of post-surgery wound infection include a wound with separated or gaped open edges accompanied by discharges such as serous, purulent, or bloody discharge, redness, inflammation, or swelling around the wound, and pain and fever. These symptoms should be observed within the first 30 days (one month) after surgery or within a year after surgery in patients receiving implants.	Australian Surgical Site Infections Surveillance (Australian Commission on Safety and Quality in Health Care, 2017).
Hospital-acquired pneumonia	Productive cough with purulent sputum (off-white, yellow, green, or opaque sputum), fever, tachypnoea (abnormal rapid and difficult breathing), shortness of breath, and white blood cell count abnormalities occurring 48 h after admission to the hospital. In addition to these syndromes, ventilator-associated pneumonia is typically associated with increased tracheal secretions and worsening oxygenation in patients who have been on mechanical ventilation for more than 48 h.	International Medical Centre of Japan Hospital syndromic surveillance for early detection of a nosocomial outbreak of acute respiratory infection (Kawana et al., 2006).
Hospital-acquired diarrheal illnesses	Frequent passage of watery stool with abdominal cramps, nausea, vomiting, blood in stool, and fever, occurring 48 h after hospital admission.	Saskatchewan *Clostridium difficile* Infection Surveillance Protocol (Domer et al., 2021), and European surveillance of Clostridioides (Clostridium) difficile infections (Nabi et al., 2020).
Catheter-associated urinary tract infection (CAUTI)	Bladder sensation, urgency, frequency, dysuria, pain in the urinary tract, suprapubic tenderness, fever, rigors, altered mental status, malaise, lethargy with no other identified cause, flank pain, and/or costovertebral angle tenderness occurring in patients with current or history of catheterization in the last 48 h.	Agency for Healthcare Research and Quality surveillance protocols for CAUTI (Baugh et al., 2011) and National Healthcare Safety Network surveillance protocol for healthcare-associated urinary tract infection (Hope et al., 2008).
Hospital-acquired bloodstream infections	Signs and symptoms of hospital-acquired bloodstream infections include fever, chills, rapid heart rate, low blood pressure, fatigue, confusion, and redness, swelling, or pain at the catheter insertion site. Signs and symptoms of sepsis include rapid breathing with high-grade fever, rapid heart rate, confusion (altered mental state), and low blood pressure in some instances, occurring 48 h or more after hospital admission.	Surveillance of healthcare-associated infections and prevention in European intensive care units (King et al., 2004).

### Surgical site infections

Surgical site infections (SSIs) are infections that occur within the first 30 days (one month) after surgery or within a year after surgery in patients receiving implants that affect either the skin, muscles, tissues or even organs at the operation site (Padilla et al., 2019; Owens and Stoessel, 2008; Smyth and Emmerson, 2000). Wound discharges (serous, purulent, and bloody), redness, inflammation or swelling around the wound, pain, fever, and separating or gaping wound edges are significant indicators of SSIs within the first 30 days post-surgery (Nasser et al., 2013). These commonly associated signs and symptoms should be prioritized in the syndromic surveillance of SSIs (Monahan et al., 2020; Costabella et al., 2023).

### Hospital-acquired pneumonia and acute lung injury

These are usually suspected if a patient develops new signs and symptoms consistent with lower respiratory infections occurring 48 h after admission to the hospital (Russell et al., 2016). These signs and symptoms include productive cough with purulent sputum (off-white, yellow, green, or opaque sputum), fever, tachypnoea (abnormal rapid and difficult breathing), shortness of breath, changes on chest X-rays, and white blood cell count abnormalities (Russell et al., 2016). Ventilator-associated pneumonia (VAP) is the most prevalent type of hospital-acquired pneumonia, with a global incidence of 15.6% and occurring at a rate of 8–28% (10–41.5 per 1,000 ventilator days) in LMICs (Xie et al., 2018; Mazwi et al., 2023). VAP occurs in patients who have been on mechanical ventilation for more than 48 h with associated risk factors including patient characteristics such as being over 65 years old, male sex, smoking, and prolonged mechanical ventilation. Other factors include disorders of consciousness, head trauma, burns, and various comorbidities such as coronary heart disease, diabetes, pre-existing pulmonary disease or chronic obstructive pulmonary disease, HIV infection, and multiple organ system failure. Furthermore, the risk of developing VAP is heightened by prior antibiotic therapy, invasive procedures, and certain gene polymorphisms (Mazwi et al., 2023).

### Hospital-acquired diarrheal illnesses

Hospital-acquired diarrheal infections are a significant concern, affecting patients after 48 h of hospitalisation or admission. These infections manifest through several signs and symptoms, such as abdominal cramps, increased stool frequency and consistency (watery stool with mucus), nausea or vomiting, bloody stool, and even fever in some instances (Bhuiyan et al., 2014). The leading cause of hospital-acquired diarrheal illnesses worldwide is *Clostridium difficile* (CD), with its incidence and severity having risen in recent years (European Centre for Disease Prevention and Control, 2017). It typically affects patients who have been treated with antibiotics, which can disrupt the normal balance of bacteria in the gut, allowing *C. difficile* to proliferate and cause infection. This infection can lead to symptoms ranging from mild diarrhoea to severe, life-threatening colitis. The onset of CD in hospitals is facilitated by factors such as advanced age, female sex, admission from a long-term acute care facility, immunosuppression, length of hospital stay, and exposure to certain classes of antibiotics (Watson et al., 2018).

### Catheter-associated urinary tract infection

Catheter-associated urinary tract infection (CAUTI) is the most prevalent HCAI and a leading cause of secondary bloodstream infections globally (Werneburg, 2022). CAUTI typically occurs in a patient whose urinary bladder is currently catheterized or has been catheterized within the past 48 h. Approximately 12 to 16% of adult hospital inpatients will have an indwelling urinary catheter (IUC) at some point during their hospital stay. Each day the IUC remains in place, the patient’s risk of developing a catheter-associated urinary tract infection increases by 3 to 7% (Magill et al., 2018). The burden of CAUTI in Africa remains significant and is on the rise in sub-Saharan Africa (Asmare et al., 2024a,b; Musinguzi et al., 2019). Symptoms and signs of CAUTI include increased bladder sensation, urgency, frequency, dysuria, pain in the urinary tract, suprapubic tenderness, fever, rigors, altered mental status, malaise, lethargy with no other identified cause, flank pain, and/or costovertebral angle tenderness and risk factors include age, female gender, diabetes, and prolonged catheterization time (Werneburg, 2022).

### Hospital-acquired bloodstream infections

Hospital-acquired bloodstream infections (HA-BSI) occur when pathogens enter the bloodstream during a hospital stay, often due to invasive procedures such as central and peripheral vascular line insertions and the associated inadequate infection prevention and control practices. The majority of HA-BSI cases are vascular catheter-related, including central line-associated bloodstream infections and peripheral vascular catheter-related bloodstream infections (Gahlot et al., 2014). However, primary HA-BSIs can also occur in the absence of identifiable sources such as indwelling catheters, requiring comprehensive clinical algorithms that integrate detailed medical history, thorough physical examination, and continuous evaluation and monitoring of clinical signs of infection for accurate identification and diagnosis (Long et al., 2022). With a high mortality rate of up to 42%, HA-BSI pose a significant global burden, particularly affecting patients in intensive care units (ICUs) (Tabah et al., 2023). Signs and symptoms of hospital-acquired bloodstream infections include fever, chills, rapid heart rate, low blood pressure, fatigue, and confusion. Patients may also experience localized symptoms such as redness, swelling, or pain at the catheter insertion site (Gahlot et al., 2014). Sepsis is a life-threatening outcome of HA-BSI, commonly known as hospital-acquired sepsis, and is frequently encountered in high-risk patients in ICUs (Tabah et al., 2023). Sepsis develops when the body’s response to an infection spirals out of control, damaging its own tissues and organs. Several signs and symptoms have been found to be commonly associated with sepsis, including rapid breathing, high fever, rapid heart rate, confusion (altered mental state), and, in some instances, low blood pressure (Page et al., 2015; Drewry et al., 2013).

## The additional role of emergency department syndromic surveillance systems for HCAIs in resource-limited settings

HCAIs have been found to be a major threat to patient safety globally, particularly those in emergency departments (Haque et al., 2020; Domer et al., 2021; Nabi et al., 2020), the first point of contact for patients requiring acute care or with injuries in most health facilities (Baugh et al., 2011). Traditional HCAI surveillance methods, which rely on laboratory confirmation and have significant turnaround times, face major limitations in the emergency departments of facilities in resource-limited settings. Early detection of potential HCAI and outbreaks, broad coverage over a wide range of syndromes, and timely collection and analysis of patient syndrome data for quicker response are critical advantages that make emergency department-based syndromic surveillance a preferable approach for HCAI surveillance in resource-limited settings (Hope et al., 2008; King et al., 2004; Hughes et al., 2020; Wu et al., 2008).

If well streamlined, the emergency department-based syndromic surveillance can be scaled up to health facility networks and eventually nationwide networks, standardizing HCAI data collection for facilitating comparison and national-level analysis (Wu et al., 2008). These networks would facilitate resource sharing and exchange of expertise and best practices for implementing and maintaining syndromic surveillance systems. They would also allow real-time analysis of syndromic data, enabling faster identification and response to potential outbreaks across facilities and regions. In the long run, the data would provide valuable insights into HCAI trends, informing public health policy and resource allocation for prevention and control. However, establishing these networks requires careful consideration of factors such as data accuracy, completeness of patient information, capacity for data interpretation, non-specificity, distinguishing between HCAIs and other infections, development of standardised data collection tools, and ensuring the necessary information technology infrastructure is in place (Government of Saskatchewan, 2016).

## Recommendations

Maximisation of emergency department-based syndromic surveillance systems for HCAIs in resource-limited settings requires strategic, tailored interventions and approaches. Standardisation of data collection forms to simplify syndromic data collection is crucial, as it alleviates the burden on already strained healthcare resources (Hughes et al., 2020). These standardised forms, whether paper-based or electronic, should be designed to require minimal time, capacity, and effort from healthcare workers within emergency departments. Additionally, they should also be interoperable with existing infrastructure, such as health facility information and record systems, to ensure streamlined data collection, accuracy and timeliness (Mandl et al., 2004).

The development and rolling out of guidelines and a comprehensive curriculum for emergency department-based syndromic surveillance of HCAIs are critical steps in fostering a culture of proactive identification, detection, and response among healthcare workers. The guidelines should include simplified case definitions for specific priority syndromes relevant to the setting or health facility. The curriculum should be straightforward yet comprehensive, to equip healthcare professionals with the skills to recognize and appropriately report potential HCAI syndromes. Enhancing capacity for emergency department-based syndromic surveillance will also secure leadership and governance support, ensuring the sustainability of the program or system.

It is critical to establish real-time reporting of syndromic data from emergency departments of health facilities to public health authorities. This involves developing clear reporting guidelines, communication channels, and automated systems to streamline data transmission. Enhancing data analysis systems based on these automated systems will enable quick analysis of HCAI syndrome data, facilitating the detection of abnormal patterns or spikes indicative of potential outbreaks. Clear and concise algorithms, along with visualisation tools, should be developed to support this process.

Fostering collaboration and data sharing among health facilities, public health agencies, and relevant stakeholders is fundamental for effective syndromic surveillance. Enhanced data and information sharing and coordination of response efforts strengthen the surveillance system through networks and partnerships and improve access to technical expertise and other shared resources (Nsubuga et al., 2011).

As the syndromic surveillance system and networks are established, continuous monitoring and evaluation of the activities—including data quality, timeliness, and system impact—are crucial for refining and adapting the system (Hughes et al., 2020). This continuous monitoring ensures that the unique challenges and characteristics of the system and local context, like healthcare infrastructural limitations, cultural practices and prevalent HCAIs, are identified early to tailor syndromic surveillance guidelines and response strategies accordingly.

## Limitations and challenges of syndromic surveillance systems for HCAIs in resource-limited settings

Syndromic surveillance, while a promising approach for the early detection of HCAIs in resource-limited settings, it faces various challenges and limitations. The systems rely on the detection and interpretation of symptoms, which can lead to misclassification, false positives, and missed diagnoses. Symptoms of HCAIs, such as fever or respiratory distress, often overlap with many infectious and other non-infectious conditions. This is significant in resource-limited settings, where the prevalence of comorbidities, such as non-communicable diseases, may be underestimated or overlooked (Kamvura et al., 2022), complicating accurate diagnosis and reducing the system’s effectiveness. In such cases, the inclusion of laboratory findings becomes essential for accurate diagnosis. Effective detection and interpretation of symptoms of HCAIs requires thorough clinical skills. However, healthcare professionals in resource-limited settings often face heavy workload and time constraints, lack of specialized training, and limited access to diagnostic tools (AbdulRaheem, 2023), increasing the risk of misdiagnosis or delayed identification of HCAIs. Enhancing training for healthcare workers to recognize a wider range of conditions, alongside improving diagnostic support, can help mitigate these challenges and improve the accuracy of syndromic surveillance systems.

Another significant limitation is the common practice of self-medication and delayed presentation to healthcare facilities. In many LMICs, patients often seek over-the-counter treatments for symptoms such as fever or cough, which can mask or alter the clinical presentation of HCAIs. This makes it more difficult for healthcare professionals to accurately diagnose infections based solely on symptoms. Public health education campaigns to raise awareness about the risks of self-medication and the importance of seeking timely medical care, and strengthening community-based health services, including outreach programs and early symptom screening can help mitigate this.

Syndromic surveillance systems for HCAIs, though potentially cost-effective, still require resources for implementation and maintenance. In resource-limited settings, competing health priorities and limited funding may restrict the development and sustainability of such systems. Without adequate financial support, the necessary infrastructure—such as digital tools for real-time data collection or analysis platforms—remains insufficient.

Additional limitations and challenges include the lack of specificity in syndrome definition, data quality concerns (Yoon et al., 2017), time lag between symptom onset and diagnosis, and constraints in the granularity of the data (Todkill et al., 2016). Reporting and selection biases also affect data quality, and it may not be sensitive enough to detect low-incidence infections (Todkill et al., 2016).

## Conclusion

Healthcare-associated infections are a significant public health threat, particularly in resource-limited settings. This narrative review advocates for the use of syndromic surveillance to identify potential HCAIs, highlighting its advantages, such as early warnings of possible outbreaks, reduced costs, higher sensitivity leading to better estimation of the burden of HAIs and reduced strain on laboratory resources. Effective emergency department-based HCAI syndromic surveillance relies on the consistent collection of quality data, comprehensive healthcare worker training, real-time reporting, stakeholder collaboration, and ongoing monitoring and evaluation for continuous improvement. Implementing this approach can significantly enhance the capacity to combat HCAIs and improve patient safety in resource-limited settings.
